# A generalized least-squares framework for rare-variant analysis in family data

**DOI:** 10.1186/1753-6561-8-S1-S28

**Published:** 2014-06-17

**Authors:** Dalin Li, Jerome I Rotter,, Xiuqing Guo

**Affiliations:** 1 Medical Genetics Institute, Cedars-Sinai Medical Center, Los Angeles, CA 90048, USA; 2 David Geffen School of Medicine, University of California Los Angeles, Los Angeles, CA 90095, USA; 3 Institute for Translational Genomics and Population Sciences, Los Angeles Biomedical Research Institute and Department of Pediatrics, Harbor-UCLA Medical Center, Torrance, CA 90502, USA

## Abstract

Rare variants may, in part, explain some of the hereditability missing in current genome-wide association studies. Many gene-based rare-variant analysis approaches proposed in recent years are aimed at population-based samples, although analysis strategies for family-based samples are clearly warranted since the family-based design has the potential to enhance our ability to enrich for rare causal variants. We have recently developed the generalized least squares, sequence kernel association test, or GLS-SKAT, approach for the rare-variant analyses in family samples, in which the kinship matrix that was computed from the high dimension genetic data was used to decorrelate the family structure. We then applied the SKAT-O approach for gene-/region-based inference in the decorrelated data. In this study, we applied this GLS-SKAT method to the systolic blood pressure data in the simulated family sample distributed by the Genetic Analysis Workshop 18. We compared the GLS-SKAT approach to the rare-variant analysis approach implemented in family-based association test-v1 and demonstrated that the GLS-SKAT approach provides superior power and good control of type I error rate.

## Background

Rare variants may, in part, explain some of the missing heritability in current genome-wide association studies [1]. Many rare-variant analysis approaches have been proposed in recent years [2-9]; however most are aimed at population-based case-control samples. Because most of the rare variants arise from recent mutations in pedigrees [10], the family-based design has the potential to enhance our ability to enrich for rare risk or protective variants that occur in the pedigrees over several generations and can substantially increase power. We recently developed an analysis strategy based on generalized least squares (GLS) [11] for family-based rare-variant association analysis, in which we first use the kinship matrix to decorrelate the family-based data, then apply a SKAT-O [9] approach to the decorrelated data, which we term the GLS-SKAT approach (Li D, personal communications, 2013). In this study, we applied the GLS-SKAT method to analyze the simulated systolic blood pressure (SBP) data in the family sample provided by the Genetic Analysis Workshop 18 (GAW18) to examine its performance.

## Methods

### Residual calculation for SBP in GAW18 simulated data

Independent individuals in the GAW18 data were extracted based on the list of unrelated individuals provided by the GAW18 organizers. For each simulation, a linear regression model was built based on the independent individuals using covariates including gender, age, age square, antihypertension medicine usage, and smoking. The linear regression model built from the unrelated subjects was then projected to the correlated subjects and the residuals were calculated for all individuals in GAW18.

Because the GLS-SKAT approach (Li D, personal communications, 2013) can implicitly control for population substructure, neither ethnicity nor principal components were included as covariates in the residual calculation.

### Kinship matrix calculation

The software EMMAX [12] was used for kinship matrix calculation. To ensure that the kinship matrix was nonsingular, one individual in each identical twin pair was excluded. Variants with minor allele frequencies (MAFs) less than 0.01 were also excluded in the kinship matrix calculation.

### GLS transformation

GLS transformation was performed using R code developed in-house. The GLS transformation has been described in detail elsewhere (manuscript in preparation). Briefly, a transformation matrix was calculated as the inverse of the decomposition of the kinship matrix. Then, this transformation matrix was used to decorrelate the family data by multiplying it with both the phenotype and genotypes matrices. The residual of SBP after controlling for the covariates was used as the phenotype. Given the time limitation, only simulated chromosome 3 genotype data was used as recommended by the GAW organizer. All variants, both common and rare, were transformed in this step.

### Analysis of the decorrelated data

For gene-based inference, the SKAT-O approach [9] was applied to the decorrelated data. Here, a gene region was defined as 20 kilobases (kb) up and downstream of the gene transcript start and stop sites, respectively. Both rare and common variants were included in this analysis, and the single-nucleotide polymorphisms (SNPs) were weighted inversely to their MAF based on the weighting framework previously proposed by Bowling and Bowling [4]. Again, no population stratification measures nor ethnicity information was included.

This GLS-SKAT procedure was applied to replicates 1 to 100 in the GAW18 simulated data. Power and type I error rate were calculated based on the 100 simulated data sets. To achieve a family-wise error rate of 0.05 after applying the Bonferroni correction, an alpha level of 4.0E-5 was utilized in the power calculation to account for the 1247 gene regions on chromosome 3.

### Family-based association test for rare variants

As a comparison we analyzed the same 100 data sets using the family-based association test (FBAT) test for rare variants (described in the updated FBAT version 2.04). There are 2 versions of the FBAT test for rare variants: FBAT-v0, which weights all the variants equally, and FBAT-v1, which weights the variants inversely to their MAF. Here we used the FBAT-v1 in the analysis. For consistency, both rare and common variants also were analyzed via the FBAT-v1 test.

We were blind to the GAW18 answer sheet when we carried out the analyses. We compared the results to the answer sheet and carried out power calculations only after the GAW18 meeting.

## Results

Detailed description of the GAW data can be found in the work of Almasy et al [13]. In the GLS-SKAT analysis, 2 individuals who were identical twins of others were excluded in the kinship matrix calculation, leaving 847 individuals with both phenotype and genotype data in the analysis. In the FBAT-v1 analysis, 369 trios from the GAW18 data were used because the FBAT test can only use trios.

**Figure 1:**
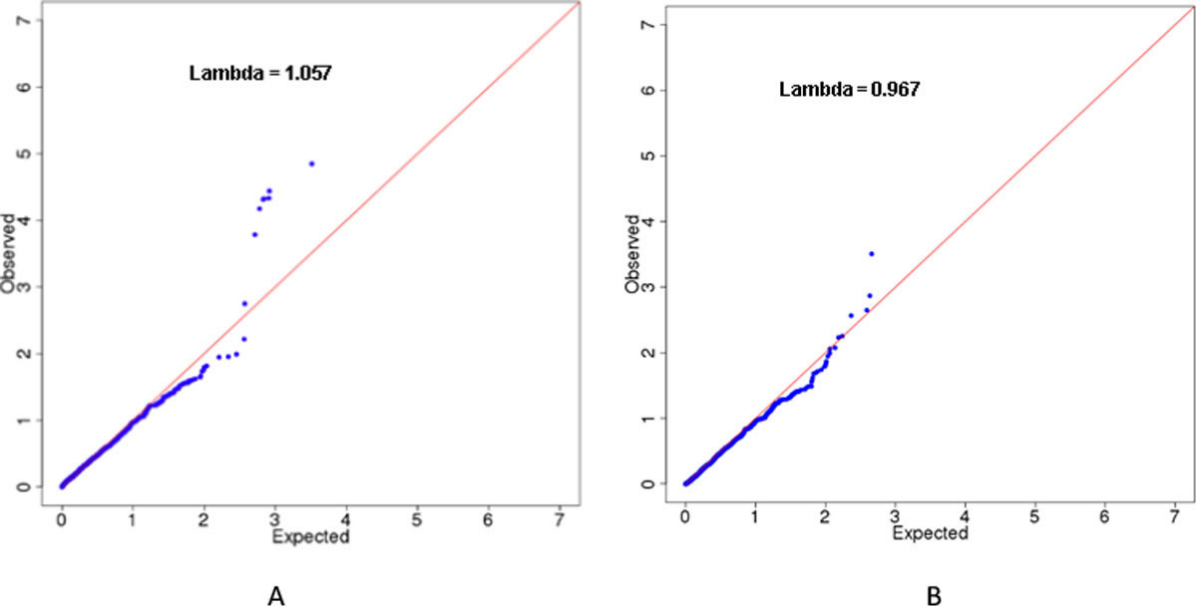
**QQ plot when applied to the first simulation of the GAW18 SBP data**. Analysis was performed using the simulated SBP data in GAW18 after controlling for gender, age, age^2^, antihypertension medicine usage, and smoking. Only chromosome 3 data were used in this analysis. The figures show the QQ plots for GLS-SKAT and FBAT-v1 analyses in the first simulation. (A) QQ plot for the GLS-SKAT analysis. (B) QQ plot for the FBAT-v1 analysis.

Figure 1 shows the QQ plots of SBP analysis results in the first simulation (chromosome 3 only) for both GLS-SKAT and FBAT-v1 methods. The lambda of the QQ plot is 1.057 for GLS-SKAT and 0.967 for FBAT.

To calculate the type I error rate, we pooled the statistics across all of the 100 simulated data sets after excluding genes located within 1 megabit (Mb) to any of the causal SNPs on chromosome 3. The type I error rate was calculated for both the GLS-SKAT and FBAT-v1 analyses (Table 1). Both approaches have type I error rates close to the nominal alpha level.

**Table 1:** Type I error rate of the GLS-SKAT and FBAT-v1 analysis

	Alpha level		
	
	0.05	0.01	0.001
**GLS-SKAT**	0.053	9.84 × 10^−3^	1.07 × 10^-3^
**FBAT-v1**	0.051	1.05 × 10^−2^	1.02 × 10^−3^

All the genes within 1 Mb of the simulated locus (*MAP4*) were excluded in this analysis; type I error rate was calculated with test statistics for all 100 simulations.

We also calculated power using the 100 simulation data sets for both the GLS-SKAT and FBAT-V1 methods for the *MAP4 *gene region, which was simulated to contribute to SBP variations (Table 2). We observed a clear advantage to the GLS-SKAT approach, which had a power of 0.69 in comparison to 0.43 for FBAT-v1 at an alpha significance level of 0.001, and 0.34 in comparison to 0.08 with a more stringent significance level of 4.0E-5, with the correction for the number of genes tested.

**Table 2:** Power of the GLS-SKAT and FBAT-v1 analysis for the *MAP4 *gene region

	Alpha level			
	
	0.05	0.01	0.001	4.0 × 10^−5^
**GLS-SKAT**	1	0.92	0.69	0.34
**FBAT-v1**	0.98	0.83	0.43	0.08

Power was calculated with test statistics of the MAP4 gene region for all the 100 simulations.

## Discussion

We applied the GLS-SKAT approach in the simulated GAW18 data set. This approach is based on a GLS framework developed for family-based samples, in which the kinship matrix is first computed using the high-dimensional genetics data, and then the kinship matrix is used to decorrelate the family data. Building upon the belief that the property of this framework would lead to a data projection that would yield a best unbiased linear estimate for each single variant in the genome (paper in preparation), we have followed the SKAT-O approach [9] for gene-/region-based inference to capture the potential causal rare variants.

Application of the GLS-SKAT approach to GAW18 simulated family data showed that the GLS-SKAT approach does not have inflated type I error rates with complex family structures, indicating that the GLS-based framework can properly decorrelate family data. Moreover, in all analyses when using GLS-SKAT, this was observed without incorporating any covariates related to ethnicity or population substructure, indicating that, by accounting for the pairwise relatedness, the kinship matrix can be used to control for both major scale population structure (ie, ethnicity) and the much finer scale substructures (ie, family structure). This is consistent with what has been observed in previous investigations [12,14].

By incorporating all family members in the analysis, the GLS-SKAT approach makes full use of all phenotype and genotype data. This is an advantage compared to the FBAT-based test, in which only trios were included. Moreover, because the FBAT-based analysis is based on transmission disequilibrium, the phenotypes of parents do not contribute to the test statistics. This leads to additional loss of information and consequently lower power, as observed in this study.

Another advantage of the GLS-SKAT approach is that it does not rely on the known family structure because the kinship matrix is estimated using the high dimensional genetics data, making it robust to the potential errors of unidentified cryptic family structure. This is particularly important for samples with complex large pedigrees, in which the cleaning and correction of the self-reported family structure using genetic data are cumbersome.

The GLS approach is similar in spirit to the previously proposed EMMAX and GRAMMAR approach [12,14], as well as the recently proposed GRAMMAR-GAMMA approach [15], because all those methods use the kinship matrix or covariance matrix to remove the influence of family structure. In particular, the GRAMMAR-GAMMA approach by Svishcheva et al [15] proposed to transform the phenotype vector using the covariance matrix to speed up the score-based association test. Our GLS approach is based on a very similar idea except that we propose to transform both phenotype and genotype, which allows us to completely ignore the covariance matrix when calculating the test statistics. This should lead to additional computational efficiency and potentially fewer restrictions in practice.

In the application of the GLS approach to GAW18 data, the transformation was performed using the kinship matrix after controlling for all covariates that contribute to SBP in the simulation model. However, in practice there could be residual correlation as a result of the structure of unknown covariates within the family, as one reviewer pointed out. This potential problem can be solved by using a covariance matrix instead of the projection matrix in the GLS transformation, in which the covariance matrix is a weighted average of the kinship matrix and an identical matrix with the weight being the calculated hereditability. This is similar to the approach used in GRAMMAR-GAMMA [15].

## Conclusions

In summary, we applied the GLS-SKAT approach to GAW18 simulated SBP data. The results demonstrated that the GLS-SKAT approach can properly control for both population and family structure using the kinship matrix estimated from high dimensional genetics data. By using all the available phenotype and genotype information, we demonstrated that the GLS-SKAT approach has a clear advantage in terms of power compared to the FBAT-based test.

## Competing interests

The authors declare that they have no competing interests.

## Authors' contributions

DL conceived and performed the analysis and drafted the manuscript. JIR and XG supervised the whole study and helped to prepare the manuscript. All authors read and approved the final manuscript.
